# Targeting the PI3K/AKT/mTOR/NFκB Axis in Ovarian Cancer

**DOI:** 10.33696/immunology.1.022

**Published:** 2020

**Authors:** Alia Ghoneum, Ammar Yasser Abdulfattah, Neveen Said

**Affiliations:** 1 Department of Cancer Biology, Wake Forest University School of Medicine, and Comprehensive Cancer Center, Winston Salem, NC 27157, USA; 2 Department of Pathology, Wake Forest University School of Medicine, and Comprehensive Cancer Center, Winston Salem, NC 27157, USA; 3 Department of Urology, Wake Forest University School of Medicine, and Comprehensive Cancer Center, Winston Salem, NC 27157, USA; 4 Wake Forest Baptist Health Sciences, Winston Salem, NC 27157, USA

**Keywords:** PI3K/AKT/mTOR/NFκB, Ovarian cancer, Therapeutic implication

## Abstract

Ovarian cancer stands as the most lethal gynecologic malignancy and remains the fifth most common gynecologic cancer. Poor prognosis and low five-year survival rate are attributed to nonspecific symptoms at early phases along with a lack of effective treatment at advanced stages. It is thus paramount, that ovarian carcinoma be viewed through several lenses in order to gain a thorough comprehension of its molecular pathogenesis, epidemiology, histological subtypes, hereditary factors, diagnostic approaches, and methods of treatment. Above all, it is crucial to dissect the role that the unique peritoneal tumor microenvironment plays in ovarian cancer progression and metastasis. This short communication seeks to underscore several important aspects of the PI3K/AKT/mTOR/NFκB pathway in the context of ovarian cancer and discuss recent advances in targeting this pathway.

## Phosphoinositol 3 Kinase (PI3K)

Phosphoinositol 3 kinase (PI3K) defines a class of lipid kinases that have the ability to phosphorylate the inositol ring 3′-OH group in inositol phospholipids and therefore produce phosphatidylinositol (3,4,5)-trisphosphate (PIP3) [1]. PI3K encompasses a family of enzymes divided into: Class IA PI3K which includes three isomers (α, β, δ) and Class IB which include the group (γ) [2,3]. PI3K Class IA is comprised of a regulatory subunit p85 along with a catalytic p110α subunit [4]. Mutations in the gene encoding the catalytic subunit of PI3K p110α, *PIK3CA*, are found in nearly 33% of clear-cell carcinoma cases [5,6], 20% of endometrioid and clear-cell carcinomas [7], 18–28% of cases of serous cystadenocarcinoma, with enhancement of the signature of activated PI3K in the majority of ovarian cancer cases irrespective of the subtype [8]. *PIK3CA* mutations are considered driver mutations that provide transformative advantages for high grade serous cancer (HGSC) [9]. Multivariate survival analysis revealed that PI3K protein expression was associated with poor survival in advanced HGSC [10]. In addition, several studies have shown that the rate of mutations in the PI3K pathway, especially in AKT and p70S6K, including missense mutations and amplifications, is correlated with higher rates of chemoresistance [11,12]. Chemo-sensitization could be achieved via downregulation of PI3K and/or its downstream effectors, AKT and mTORC1 [13–15]. The increased activation of PI3K in OvCa and its role as a hub for several cancer-promoting pathways, explain its many implications in cancer progression including oncogenic transformation, cell proliferation, adhesion, and apoptosis, as well as multiple metabolic pathways, thus aptly positioning it as a target for therapeutic advancement [16–19].

## Protein Kinase B PKB/AKT

The AKT/PKB family comprises a group of serine threonine kinases, which are cAMP- and cGMP-dependent [20]. Three AKT isoforms have been identified: AKT1 (PKBα), AKT2 (PKBβ), and AKT3 (PKBγ) [1,21]. AKT1 is involved in cellular growth, angiogenesis, and tumor cell invasiveness. AKT is the main kinase which integrates upstream signals from PI3K and mammalian target of rapamycin complex 2 (mTORC2) with downstream signals to mTORC1 with subsequent activation of downstream substrates that induce cell cycle progression, protein synthesis, and cell growth [21], and dictate several cellular activities such as survival, proliferation, and migration [18,20,22]. Moreover, AKT promotes protein synthesis and cell growth through inhibition of tuberous sclerosis complex 2 (TSC2), and 4E-binding protein 1 (4E-BP1), that inhibit cell growth in various cancer types, and regulate mRNA translation and cellular proliferation, respectively [17,21,23–25]. AKT is inhibited by tumor suppressors including phosphatase and tensin homolog (PTEN) and inositol polyphosphate 4-phosphatase type II (INPP4B). In ovarian cancer, AKT1 is mutated and AKT2 is amplified in about 40% [17,26]. Overexpression of AKT in OvCa is associated with advanced stage-platinum resistance [12,27]. Furthermore, data curated from The Cancer Genome Atlas (TCGA) revealed that the expression of AKT1, AKT2, and AKT3 was associated with poor patient survival [28].

## Mammalian Target of Rapamycin (mTOR)

mTOR comprises two biochemically and functionally independent catalytic complexes, mTORC1 and mTORC2. Both mTOR complexes are implicated in the induction of angiogenesis, proliferation, and cellular survival [2,29]. Phospho-mTOR activates two downstream targets: 4E-binding protein 1 (4E-BP1) and ribosomal protein S6 kinase (S6K). In aggressive cancers, 4E-BP1 functions as a hypoxia-inducible switch, allowing for translation of factors, and hence facilitating angiogenesis and anti-apoptotic cell growth [25,30]. Phosphorylated S6K is required for cell growth and G1 cell cycle progression [31,32]. mTORC1 is activated and overexpressed along with its downstream effectors, 4EBP1 and p70S6K, in advanced HGSC [8,33] warranting the use of mTOR inhibitors as targeted therapies in several clinical trials [34–37]. Consistently, analysis of TCGA data indicated that high expression of mTOR is associated with poor survival rates in patients with advanced stage HGSC.

## Nuclear factor-κ light chain enhancer of activated B cells (NFκB)

Nuclear factor-κ light chain enhancer of activated B cells (NFκB) encompasses a group of transcription factors that are divided into two classes: Class I, which include NFκB1 or p50/p105, and NFκB2 or p52/p100. Class II includes RelA/p65, RelB, and c-Rel [38,39]. The NFκB canonical pathway includes NFκB, IkBα, and RelA/p65. IkBα is phosphorylated by the Inhibitor of Nuclear Factor Kappa B Kinase (IKK) [40,41]. Upon phosphorylation of NFκB1, the IkBα subunit undergoes ubiquitination and subsequent proteasomal degradation. This allows p50 and p65/RelA to dimerize and translocate to the nucleus where the heterodimer induces transcription of genes involved in inflammation, cell growth, chemoresistance, and apoptosis [42–44]. Alternatively, the non-canonical pathway is activated when inflammatory cytokines, TNF and IL1, bind to their respective receptors and subsequently signal to NFκB inducing kinase (NIK) to activate the IKK complex [45]. High expression of the p65/RelA subunit of NFκB, along with cleaved caspase 3 confers poor outcomes in OvCa patients [46]. Jinawath et al., [44] demonstrated that inhibition of NFκB resulted in enhanced efficacy of cisplatin *in vitro* and *in vivo* OvCa models. Upregulation of the p65/RelA subunit of NFκB increased the resistance of OvCa to carboplatin [44], and significantly enhanced the aggressiveness of OvCa cells [47]. Our earlier report [28] showed that in HGSC data from TCGA, the expression of NFκB subunits, p65RelA, NFκB1 and NFκB2 as well as IKKβ were associated with poor patient survival.

## PI3K/AKT/mTOR/NFκB Axis

**Figure 1:**
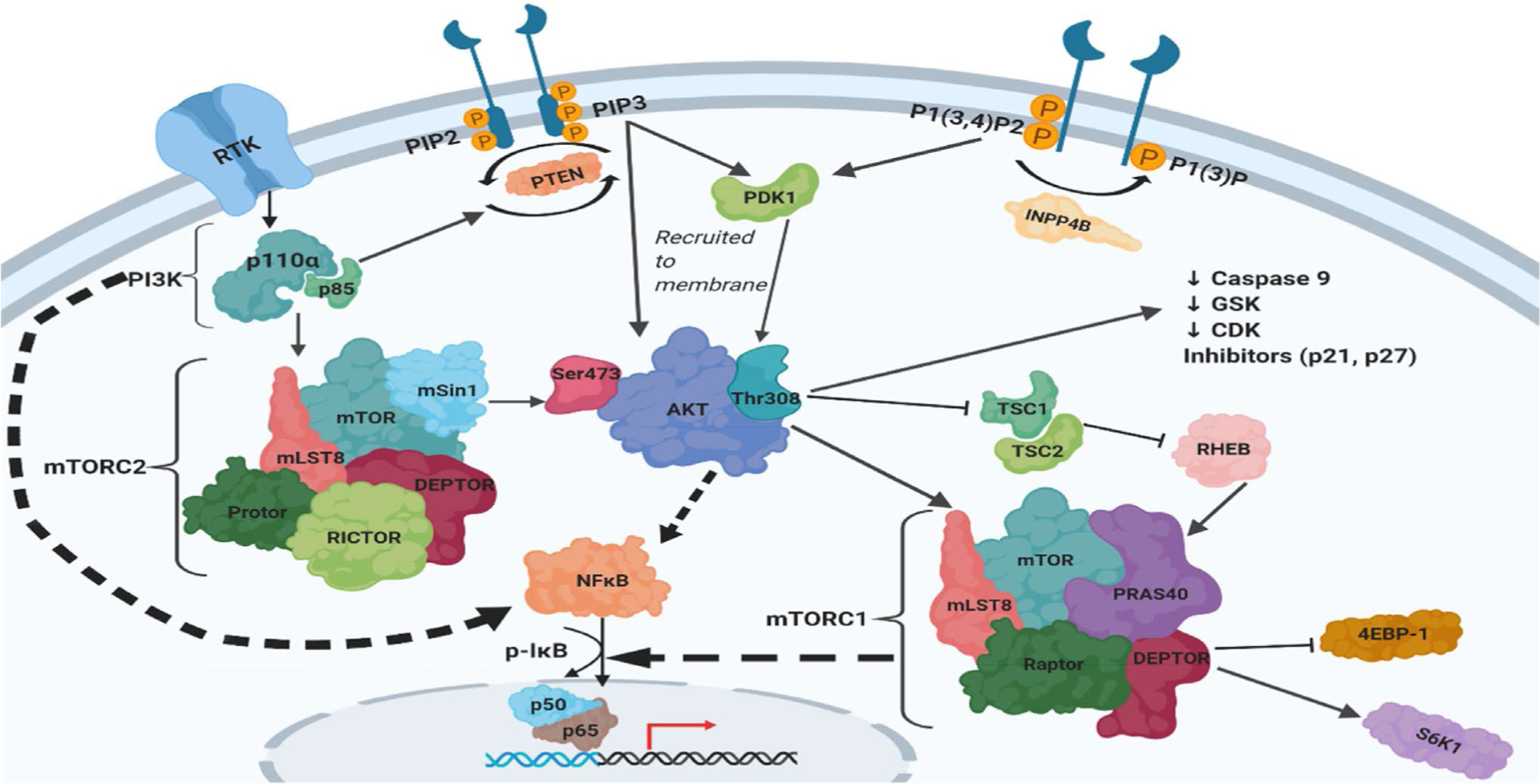
PI3K/AKT/mTOR/NFκB Pathway in Ovarian Cancer. (Created with Biorender)

The PI3K and NFκB pathways are involved in a complex crosstalk (Figure 1) which results in decreased survival rates in OvCa patients [48]. The PI3K catalytic subunit p110α and its regulatory subunit p85 have been shown to directly activate NFκB [49–51]. Overexpression of the p110α subunit induces p65/RelA activation and nuclear translocation. PI3K activation also phosphorylates AKT with subsequent activation of the p65/RelA subunit of NFκB via phosphorylation through the IKK complex. Phospho-AKT mediates the phosphorylation of IKKα allowing for it to phosphorylate IkB, and hence allowing NFκB to translocate into the nucleus [52]. Moreover, AKT can activate NFκB independently of IKK by directly phosphorylating the p65/RelA subunit [53]. Importantly, analysis of TCGA data revealed a positive correlation between the transcripts of *PIK3CA, AKT1/2/3*, as well as *NFkB subunits* [28].

## Recent Advances in Targeting PI3K/AKT/mTOR/NFκB Axis

Several therapeutics are being developed in pre-clinical models to target PI3K/AKT/mTOR/NFκB axis in ovarian cancer. A seminal study by Yoon et al., recently reported that methyl lucidone (ML) from the dried fruit of *Lindera erythrocarpa makino* (Lauraceae) exerted cytotoxic effects in the OvCa cell lines, SKOV3 and OVCAR3. Specifically, ML inhibited cell proliferation with significant cellular morphological changes, and apoptosis in SKOV3. Mechanistically, ML induced apoptosis through cleavage of caspase-3/9 and Poly (ADP-Ribose) Polymerase (PARP), allowing for the release of cytochrome C from the mitochondria, decreased expression of Bcl-2 and Bcl-xL, and prompted cell cycle arrest in the G_2_/M phase. ML also led to the repression of cyclin-A/B expression and stimulated cyclin-dependent kinase inhibitors p21 and p27. Importantly, ML exerted its inhibitory downstream effects by blocking the PI3K/AKT/mTOR/NFκB axis, manifested by significant downregulation of the levels of PI3K and phosphorylated AKT concomitant with nuclear translocation of NFκB and the total level of p-IkBα [54]. Another study reported that inhibition of YAP significantly suppressed the malignant behavior of OvCa cells, via regulation of the PI3K/AKT/mTOR pathway. Interestingly, a YAP inhibitor, peptide 17, inhibited OvCa progression by inhibiting the PI3K/AKT/mTOR pathway *in vitro* and *in vivo* [55]. In addition, Diaz-Cueto et al., [56] reported that pharmacologic inhibition of PI3K/AKT/mTOR, and ERK1/2 significantly reduced progranulin (PGRN) expression with subsequent inhibition of cell proliferation and survival in platinum-resistant TOV-21G cells [56]. Interestingly, a recent study reported that PI3K/AKT/mTOR/NFκB axis is activated by ghrelin, an endogenous ligand for growth hormone secretagogue receptor (GHSR), promoting ovarian cancer cell survival and cisplatin resistance [57]. Targeting the ghrelin/ghrelin receptor pathway by ghrelin receptor antagonist, [D-Lys3]-GHRP-6 or the PI3K inhibitor, LY294002 significantly inhibited OvCa cell survival and sensitized them to cisplatin [57].

Clinically, a phase 2 clinical trial (NCT04055649), is ongoing using an orally active small molecule dopamine receptor D2 antagonist, ONC201, in combination with paclitaxel for the treatment of patients with platinum-resistant refractory or recurrent epithelial ovarian, fallopian tube, or primary peritoneal cancer. ONC201 was originally identified as a small molecule that induces transcription of TNF-related apoptosis-inducing ligand (TRAIL) and subsequently kills cancer cells by activating TRAIL death receptors [58]. Further investigation of the mechanism of action of ONC201 revealed that it acts through dual inhibition of AKT and ERK, [59], inhibition of NFκB and STAT3 [60] as well as inhibition of PI3K/AKT/mTOR [61] in a multitude of solid and hematologic malignancies, including ovarian cancer.
